# High-Throughput Identification and Screening of Novel *Methylobacterium* Species Using Whole-Cell MALDI-TOF/MS Analysis

**DOI:** 10.1371/journal.pone.0040784

**Published:** 2012-07-12

**Authors:** Akio Tani, Nurettin Sahin, Yumiko Matsuyama, Takashi Enomoto, Naoki Nishimura, Akira Yokota, Kazuhide Kimbara

**Affiliations:** 1 Institute of Plant Science and Resources, Okayama University, 2-20-1 Chuo, Kurashiki, Okayama, Japan; 2 Faculty of Education, Mugla Sitki Kocman University, TR-48170 Kotekli, Mugla, Turkey; 3 Bruker Daltonics, K.K., 3-9 A6F, Moriya, Kanagawa, Yokohama, Japan; 4 Botanical Garden, Okayama University of Science Japan, 1-1 Ridai-cho, Okayama, Japan; 5 Faculty of Mathematics and Natural Sciences, University of Indonesia, Campus UI, Depok, Indonesia; 6 Faculty of Engineering, Shizuoka University, 3-5-1 Johoku, Kita-ku, Hamamatsu, Japan; University of Iowa Carver College of Medicine, United States of America

## Abstract

*Methylobacterium* species are ubiquitous α-proteobacteria that reside in the phyllosphere and are fed by methanol that is emitted from plants. In this study, we applied whole-cell matrix-assisted laser desorption/ionization time-of-flight mass spectrometry analysis (WC-MS) to evaluate the diversity of *Methylobacterium* species collected from a variety of plants. The WC-MS spectrum was reproducible through two weeks of cultivation on different media. WC-MS spectrum peaks of *M. extorquens* strain AM1 cells were attributed to ribosomal proteins, but those were not were also found. We developed a simple method for rapid identification based on spectra similarity. Using all available type strains of *Methylobacterium* species, the method provided a certain threshold similarity value for species-level discrimination, although the genus contains some type strains that could not be easily discriminated solely by 16S rRNA gene sequence similarity. Next, we evaluated the WC-MS data of approximately 200 methylotrophs isolated from various plants with MALDI Biotyper software (Bruker Daltonics). Isolates representing each cluster were further identified by 16S rRNA gene sequencing. In most cases, the identification by WC-MS matched that by sequencing, and isolates with unique spectra represented possible novel species. The strains belonging to *M. extorquens, M. adhaesivum, M. marchantiae, M. komagatae, M. brachiatum, M. radiotolerans,* and novel lineages close to *M. adhaesivum,* many of which were isolated from bryophytes, were found to be the most frequent phyllospheric colonizers. The WC-MS technique provides emerging high-throughputness in the identification of known/novel species of bacteria, enabling the selection of novel species in a library and identification without 16S rRNA gene sequencing.

## Introduction

The surface of plant leaves (phyllosphere) is a preferred niche for various kinds of bacteria. Healthy leaf surface contains 10^6^ to 10^7^ bacterial cells/cm^2^ of leaf, and the terrestrial leaf surface area that may be colonized by microbes is estimated to be approximately 6.4×10^8^ km^2^ [1]. Plants emit a huge amount of various volatile organic compounds, including C1 compounds such as methane [2] and methanol. The global emission of methanol from plants is estimated to be 100 Tg per year [3], [4] as a result of pectin degradation during plant cell elongation and division [5].

The community composition of phyllospheric bacteria has been studied in a number of culture-independent studies, which demonstrate that the predominant species belongs to α- and γ-proteobacteria, and are dependent on plant species (clone library method, reviewed in [6]). A recent metagenomic study showed that α-proteobacteria predominate the phyllosphere of soybean samples up to 42.8%, including *Methylobacterium* species as one of the main components. A concomitant meta-proteomic study demonstrated an abundance of the enzymes involved in methanol oxidation originated from the species [7]. Thus, methanol is unequivocally one of the important carbon and energy sources for *Methylobacterium* species inhabiting the phyllosphere.

Plant species and, more strongly, location, influence the phyllospheric *Methylobacterium* community composition [8]. Moreover, in a competition experiment, *M. extorquens* strain PA1 was reported to be a highly competitive colonizer in a model experimental system using *Arabidopsis* [9]. Many isolates that belong to the genus are known to promote plant growth, possibly by synthesizing plant hormones such as auxin [10], [11], [12] and cytokinin [13], [14], and through the activity of 1-aminocyclopropane-1-carboxylate (ACC) deaminase, which lowers ethylene levels in plants [15], [16]. Their other activities, such as siderophore production [15], nitrogen fixation [17], and calcium phosphate solubilization [18] are considered to be involved in nutrient acquisition for plants. Thus, the genus is considered to be one of the major bacteria affecting plant growth.

At the time of writing, the genus comprises 35 recognized species (http://www.bacterio.cict.fr/m/methylobacterium.html). An increasing number of descriptions for novel species in the genus, many of which were isolated from plants, have been reported. The genus contains species sharing higher than 97% [19] and even more than 98.6% [20] pairwise similarity of 16S rRNA gene sequences, rendering it difficult to differentiate the species. For precise classification, the DNA-topoisomerase gene (*gyrB*) and methanol dehydrogenase gene (*mxaF*) were also used [21], [22] as alternative molecular evolutionary markers. A cultivation-independent method, automated ribosomal intergenic spacer analysis (ARISA), has been applied to evaluate *Methylobacterium* communities of leaf samples [8]–[9] [23], revealing that there are many species of potentially new lineages.

Recent advances in mass spectrometry have shed light on the rapid and precise identification and classification of microorganisms. Whole-cell matrix-assisted laser desorption ionization-time of flight mass spectrometry (WC-MS) profiling of whole cell proteins is an emerging technology for the identification of bacteria. WC-MS-based identification can accurately resolve bacterial identity at the genus, species, and subspecies levels in some taxa [24]. The mass spectral profiles primarily represent ribosomal proteins, which are the most abundant cellular proteins and are synthesized under all growth conditions [25]. Therefore, the spectral fingerprints are reproducible even in different growth media and cell growth states [25]. Different mass spectral fingerprints enable the correct identification of unknown strains through comparison with a reference library of known strains. The fingerprints can also be used to construct a hierarchical dendrogram (cluster analysis) based on the spectral similarity [26], [27], [28].

In this study, we applied the WC-MS technique to classify and identify *Methylobacterium* isolates collected from various plant samples. For this purpose, we first optimized the analytical condition and analyzed the attribution of the detected peaks. Next, we established a method for rapid identification and clustering analysis based on the spectral similarity using the type strains, and then evaluated the relationship between similarities based on 16SrRNA gene sequences and on WC-MS spectra. Furthermore, we analyzed 213 isolates belonging to the genus *Methylobacterium* from plants with WC-MS using MALDI Biotyper software (Bruker Daltonics). Though limited, the relation between the isolates and plant species as isolation sources was analyzed, and a brief phenotypic characterization of isolates was also conducted.

## Materials and Methods

### Media for Isolation and Cultivation

The methanol medium used in this study contained 0.3 g (NH_4_)_ 2_HPO_4_, 0.1 g KCl, 0.05 g yeast extract, 0.1 mg MgSO_4_.H_2_O, 10 mL vitamin solution, 10 mL metal solution, and 5 mL methanol per liter. The vitamin solution contained 0.4 g calcium pantothenate, 0.2 g inositol, 0.4 g niacin, 0.2 g *p*-aminobenzoic acid, 0.4 g pyridoxine HCl, 0.4 g thiamin HCl, 0.2 g biotin, and 0.2 g vitamin B_12_ per liter. The metal solution contained 1.9 g CoCl_2_.6H_2_O, 1.0 g MnCl_2_.6H_2_O, 0.7 g ZnCl_2_, 0.06 g H_3_BO_3_, 0.36 g Na_2_MoO_4_.H_2_O, 0.24 g NiCl_2_.6H_2_O, and 0.02 g CuCl_2_.2H_2_O per liter. R2A medium (Difco) was also used.

### Isolation of *Methylobacterium* Species from Various Plants

Leaf samples of various plants natively grown or cultured in the area or farm field in the Institute of Plant Science and Resources, Okayama University (Kurashiki, Okayama Japan) were collected in April 2008. No specific permits were required for the described field studies. The location is not protected in any way, and the field studies did not involve endangered or protected species. A piece of each leaf sample was briefly washed with 50 mL sterile water to remove dust, and then washed vigorously with 10 mL sterile water. Next, the wash solution was spread on methanol medium containing 50 mg/L cycloheximide. Samples that were not plants were appropriately diluted or suspended in water and spread as above. After incubation at 28°C for 3 to 7 days, pink-pigmented colonies were preferentially picked up and purified by re-streaking on agar plates of the same medium. When no pink-pigmented colonies were obtained, the leaf suspension (50 µL) was inoculated in 5 ml liquid methanol medium. After cultivation at 28°C for 3 days, the appropriately diluted media were spread onto the solidified methanol medium, and pink-pigmented colonies were isolated. The names of these isolates were designated as the “z” series.

In total, 409 isolates were obtained. They were then subjected to a growth test in liquid methanol medium. Approximately half of the isolates exhibited turbidity in liquid medium, and their colonies grown on methanol solid medium were subjected to WC-MS analysis, as described below. For some isolates exhibiting poor growth on methanol medium, colonies grown on R2A solid medium were used. Almost all type strains of *Methylobacterium* species were included in the analysis. *M. thiocyanatum* JCM 10893 and *M. platani* JCM 14648 were provided by the RIKEN BRC through the National Bio-Resource Project of the MEXT, Japan. The type strains of *M. populi* [29], *M. cerastii* [30], and some that have been published but not validated, such as *“M. funariae”* [31], “*M. goesingense”* [32], and “*M. longum”* [33], were not included in our mass spectrometry analysis.

### WC-MS Procedure

A loopful of well-grown bacterial cells from the last quadrant of streaked colonies (3–7 days old, usually 5–10 mg wet weight) from methanol mineral agar or R2A agar was suspended in 300 µL of 75% ethanol. The samples could be stored at -20°C without recognizable spectral changes (data not shown). The suspension was then centrifuged at 15,000 rpm for 2 min. The supernatant was discarded completely and 50 µL of 70% formic acid was added and mixed well. Next, 50 µL of acetonitrile was added and mixed well again. One microliter of the sample was placed onto a spot of a MALDI steel target plate and dried in air. Then, 2 µL of matrix solution (saturated solution of sinapinic acid in 50% acetonitrile and 2.5% trifluoroacetic acid) was overlaid onto the sample, and the samples were dried in air.

The samples were analyzed with MALDI-TOF/MS equipped with a 50 Hz nitrogen laser (Ultraflex, Bruker Daltonics). Mass spectra were obtained using a positive linear mode in the range of mass to charge ratio (m/z) 2,000 to 20,000 with suppression 800 Da (parameter settings: ion source 1, 25 kV; ion source 2, 23.35 kV; lens, 6.35 kV; detector gain, 8.4 x). Protein standard was comprised of insulin ([M+H]^+^ = 5734.56), ubiquitin-I ([M+H]^+^ = 8565.89), cytochrome *c* ([M+H]^+^ = 12361.09 and [M+2H]^2+^ = 6181.05), and myoglobin ([M+H]^+^ = 16952.55 and [M+2H]^2+^ = 8476.77) (Bruker Daltonics). The laser shots were applied until the intensity (arbitary unit) of the highest peak reached between 6000 and 10,000 (usually 300–1000 shots).

*Escherichia coli* DH5α (a derivative of *E. coli* K12) was also used as a standard to evaluate the method. The overnight-grown cells in LB liquid medium were used to evaluate the technique, and attribution of the detected peaks to ribosomal proteins was confirmed.

### WC-MS in Different Growth Conditions

To check whether WC-MS patterns change depending on cultivation time, *M. extorquens* strain AM1 (ATCC 14718) was cultured in liquid and on solid medium of methanol medium and R2A, and the samples were analyzed every day for 2 weeks. The cells from solid cultures were treated as described above. The cells in liquid culture medium (200 mL) were collected by centrifugation (5 mL each time), washed with water, and treated as above.

### Purification of Ribosomal Protein

Ribosomal proteins from strain AM1 grown on methanol medium were purified following the method reported previously [34]. The MS spectra of ribosomal proteins were compared with the aforementioned WC-MS profile to identify the detected protein peaks based on the genome information of the strain.

### Clustering Analysis of WC-MS Profiles

The WC-MS data of the isolates were analyzed by MALDI Biotyper software (Bruker Daltonics) to construct a dendrogram based on the mass spectra, under default analysis parameters (distance measure, correlation; linkage, average). After construction of the tree, 80 strains representing each cluster were selected and used for further study. Some strains in the same branch were also selected for further characterization to confirm their identity and to demonstrate the reliability of Biotyper-based classification.

To establish a Biotyper-independent technique, we developed a method for cluster analysis based on WC-MS data. The obtained WC-MS data were imported to SpecAlign software [35], followed by spectrum smoothing (3 cycles Savitsky–Golay smoothing) and baseline subtraction. The relative intensity data were used to generate an average spectrum. The spectra aligned with the peak matching method (maximum shift, 20) were exported to a spreadsheet. The binary data (peaks showing >5% relative intensities were counted as 1 and the others as 0) of m/z 3,500 to 20,000 were used for `cluster analysis with XLSTAT software (Addinsoft, Paris). The Pearson correlation coefficient and group average method were used to generate a proximity matrix and to generate a dendrogram. The correlation between the WC-MS data matrix of the type strains of *Methylobacterium* species and that of the 16S rRNA pairwise similarity matrix were analyzed by Mantel test statistics using XLSTAT.

### 16S rRNA Gene Sequencing

The selected isolates of the representative clusters were subjected to 16S rRNA gene sequencing [36]. Sequencing was carried out using an automated DNA sequencer (model 3130; Applied Biosystems) and a ca. 1.5 kb sequence was determined. Phylogenetic analysis was carried out using SILVA Aligner [37] and MEGA5 software [38] after multiple data alignments. Genetic distances were obtained by Kimura’s two-parameter distance model [39]. Phylogenetic trees were constructed by the neighbor-joining method [40]. The robustness for individual branches was estimated by bootstrapping with 1,000 replicates [41]. Pairwise nucleotide sequence similarity values were calculated using the EzTaxon server version 2.1 [42]. The alignment gap was not considered in the similarity calculation.

Methylotrophic fungi were also isolated in this study; they were identified by 28S rRNA gene sequencing, as described previously [43].

### Physiological Properties and Phenotypic Characterization of the Isolates

The characteristics of our isolates, which were considered to be important for interaction with plants, were determined. PQQ production ability was examined as reported previously [44]. Auxin (indole acetate) was measured as reported [45] using 0.5% methanol instead of glucose. Growth was tested on nitrogen-free medium (1 g K_2_HPO_4_, 0.2 g MgSO_4_, 1 g CaCO_3_, 0.2 g NaCl, 5 mg FeSO_4_, 10 g glucose [or 5 mL methanol] per liter, and 1.5% agar; pH 7.0) to examine their growth on this medium. The presence of the nitrogenase gene (*nifH*) was surveyed by polymerase chain reaction (PCR) with nifH-a2 primer sets [46]. National Botanical Research Institute’s Phosphate (NBRIP) growth medium [47] was used for the calcium phosphate (Ca-P) solubilization assay. Siderophore production was also tested according to the published method [48]. Their utilization of sugars, and the activities of urease, DNase, and oxidase were also tested.

### Nucleotide Accession Numbers

The GenBank/EMBL/DDBJ accession numbers for the 16S and 28S rRNA gene sequences reported in this paper are listed in Table S5.

## Results and Discussion

### Isolation of *Methylobacterium* Species from Various Plants

In order to determine methylotrophic species diversity and to partially characterize *Methylobacterium*-plant association specificity, we isolated pink-pigmented methylotrophs from various plant samples (112 samples). Since we were also interested in non-*Methylobacterium* methylotrophs, many non-pigmented strains were also isolated. After the isolation of 409 strains by plating, approximately half of them did not exhibit growth in liquid methanol medium. Most of them were non-pigmented, and were therefore not used in further study (data not shown). A total of 213 methylotrophic isolates were used in further study (Table S1).

### Effect of Culture Media and Cultivation Time on WC-MS Profile

To check whether culture conditions affect the WC-MS profile, strain AM1 was cultivated under different conditions for 14 days. Figure S1 shows WC-MS patterns as gel-like images; these were created with mMass 5.0 software [49]. Even though the intensity of each peak changed according to time and medium, most of the detected peaks in these conditions were identical throughout the cultivation time. Only in the earliest cultivation time, did the patterns differ in comparison to the older ones. Most of the detected peaks in these samples, however, were also detected in older samples, although their peak intensities were different. These results suggested that the age of cells and medium composition affected the WC-MS peak intensity but did not have a strong affect on the position of detected peaks. WC-MS patterns have been reported to change according to cultivation time in *E. coli* [50] and *B. subtilis* [51]. This is due to the adaptation of cells to the changing chemical environment during cultivation. The reason why drastic changes in patterns were not observed may be that a nutrient-poor medium was used in this study. We used cells grown on solid methanol medium for 5 to 7 days–during this time, the WC-MS pattern did not change significantly.

### Identification of the Peaks Detected in WC-MS and Purified Ribosome

We evaluated our WC-MS technique using *E. coli* DH5α cells prepared as described in the Materials and Methods section. Among the 58 ribosomal proteins encoded in the *E. coli* K12 genome, 50 proteins have a molecular weight less than 20,000 Da. We could detect peaks of 30 ribosomal proteins within the range (RL7, 13, 15, 18–23, 25, and 27–36, and RS8, 10, 13–15, and 18–22), and their post-translationally modified molecular weights were identical to those previously reported [50] (data not shown). Thus, WC-MS can detect ribosomal proteins using intact cells, without the use of purified ribosome. When the cells were used, peaks that could not be attributed to the ribosomal proteins were also detected. These can be attributed to ribosomal proteins with unknown modifications or abundantly produced non-ribosomal proteins. Ribosomal proteins were modified by methionine loss according to the N-terminal rule [52], methylation, acetylation, methylthiolation, phosphorylation, and others [50]. Although the biological significance of these modifications is not yet clear, phosphorylation in many ribosomal proteins is involved in the function and regulation of protein synthesis [53]. Moreover, a C-terminal 8 amino acids loss for RL31, nine methylations for RL11, and an unknown modification for RL16 also occurs in *E. coli* [50].

Next, we compared the spectra of the purified ribosomal proteins and WC-MS, using *M. extorquens* strain AM1. Table 1 is a list of the ribosomal proteins detected in the analyses of WC-MS and the ribosome fraction. Among 54 ribosomal proteins encoded in the genome, 41 were in the analysis range (m/z 2,000–20,000). Of these, 19 (WC-MS) and 23 (purified ribosome) proteins were detected. Those with higher molecular weight were not detected effectively due to the ion suppression effect. The detected m/z was in accordance with the molecular weight of proteins following the N-terminal rule [52]. The subunits L33 and S12 were detected as methylated and β-methylthiolated forms, respectively (they are so modified in *E. coli*). Other undetected ribosomal proteins were modified in unknown forms, since we detected many other peaks that were unattributable to ribosomal proteins in the range.

**Table 1:** Ribosomal subunit proteins of *M. extorquens* strain AM1 detected by MALDI-TOF/MS.

locus tag (MexAM1_MATA1p)	gene	ribosomal subunit	amino acid length	2nd amino acid	methionine loss	Theoretical average molecular mass	WC-MS	Error (Da)	Purified ribosome	Error (Da)	Remark
2938	rpmJ	L36	41	K	no	4994.94	4993.07	−1.87	4993.95	−0.99	
2356	rpmH	L34	44	K	no	5090.12	5088.29	−1.83	5089.62	−0.50	
1700	rpmG	L33	55	A	yes	6264.60			6278.49	13.89	Methylated
3696	rpmF	L32	62	A	yes	6905.14	6904.83	−0.32	6905.05	−0.10	
2174	rpmD	L30	64	A	yes	6943.34	6943.58	0.24	6943.05	−0.28	
1521	rpmI	L35	67	P	yes	7481.02	7481.77	0.75	7481.37	0.35	
2161	rpmC	L29	71	K	no	8155.33	8156.32	0.98	8156.14	0.81	
2358	rpmE	L31	84	A	yes	9119.34	9119.97	0.63	9119.99	0.65	
4323	rpsR	S18	84	A	yes	9211.99	9208.78	−3.22	9208.06	−3.94	Acetylated 9254.00 was not observed
4855	rpmA	L27	88	A	yes	9335.88					
5345	rpsT	S20	88	A	yes	9528.32	9530.89	2.57	9529.65	1.33	
2162	rpsQ	S17	88	P	yes	9692.29	9692.72	0.44	9693.75	1.46	
4417	rpsO	S15	89	S	yes	10216.97	10218.78	1.81	10218.91	1.94	
2157	rpsS	S19	92	A	no	10231.98					
2155	rplW	L23	98	S	yes	10711.55			10713.03	1.48	
2181	rplX	L24	104	A	yes	11084.87	11089.45	4.58	11085.95	1.08	
3182	rpmB	L28	101	S	no	11209.24	11206.90	−2.34	11210.13	0.89	
2179	rpsN	S14	101	A	no	11595.65	11597.20	1.55	11597.25	1.60	
2152	rpsJ	S10	102	N	no	11607.53					
4149	rpsU	S21	105	Q	no	11667.77	11667.86	0.09	11669.52	1.75	
4404	rplL	L7/L12	125	A	yes	12617.74	12616.89	−0.85			
2176	rplR	L18	120	S	yes	12682.74			12684.87	2.13	
479	rpsP	S16	120	S	yes	13078.22	13079.70	1.48	13080.20	1.98	
2182	rplN	L14	122	I	no	13347.90					
1522	rplT	L20	121	A	yes	13496.96	13500.01	3.06	13499.52	2.57	
2148	rpsL	S12	123	P	yes	13675.27			13719.69	44.42	β-methylthiolated
2170	rpsM	S13	122	A	yes	13715.28	13716.81	1.53			
2169	rpsK	S11	129	A	yes	13735.72					
2158	rplV	L22	127	G	yes	13788.35					
4856	rplU	L21	130	F	no	13904.88					
2178	rpsH	S8	131	V	yes	14497.78					
5186	rplS	L19	134	N	no	15059.31					
2160	rplP	L16	137	L	no	15458.22					
2166	rplQ	L17	138	R	no	15535.06			15537.36	2.30	
4385	rplK	L11	149	A	yes	15643.71					
2177	rplF	L6	154	K	no	16859.58					
4324	rpsF	S6	148	P	yes	16898.14					
2739	rplM	L13	153	K	no	17174.02			17172.30	−1.72	
2173	rplO	L15	169	K	no	17367.89					
4403	rplJ	L10	172	D	no	17666.73					
2149	rpsG	S7	156	S	yes	17798.47					
2740	rpsI	S9	163	I	no	18311.14					
2175	rpsE	S5	192	A	yes	20678.70					
4321	rplL	L9	190	E	no	20949.59					
2180	rplE	L5	188	A	yes	21104.74					
2154	rplD	L4	206	K	no	22517.00					
5190	rpsD	S4	205	S	yes	23572.29					
2440	rplY	L25	228	S	yes	23718.79					
4386	rplA	L1	232	A	yes	24172.39					
2153	rplC	L3	246	R	no	26108.92					
2159	rpsC	S3	248	G	yes	27531.97					
2156	rplB	L2	278	A	yes	30095.72					
2057	rpsB	S2	349	R	no	37455.57					
55	rpsA	S1	570	S	yes	62790.44					

Within the genome database, we searched the proteins with the molecular weights of the detected masses in the ribosome fraction sample. Table S2 is a list of m/z detected in ribosomal protein fraction and the identification of proteins. Many of the peaks in the low m/z range (<5000 m/z) could not be attributed to any proteins encoded in the genome. These may contain bivalent ions (M+2H^+^) or unannotated proteins. Most of the peaks could only be attributed to hypothetical proteins, whose functions are unknown. It is possible that the peaks that were unattributable to ribosomal proteins are ribosomal proteins that are modified in unknown forms. Determination of these modifications necessitates more intensive proteomic study using separated ribosomal proteins. The modifications of ribosomal proteins in bacteria other than *E. coli* are largely uncharacterized.

We also identified the peaks detected in WC-MS using strain AM1 cells grown on methanol for 6 days (Table S3). Similarly, most of the detected peaks were unattributable to any protein or were attributable to some hypothetical proteins. Interestingly, pyrroloquinoline quinone (PQQ) synthesis protein A, cold shock DNA binding protein (CspA), protein hfq, RNA-binding host factor, and granule-associated 11 kDa protein, which were not detected in the ribosomal protein fraction, were observed as possible proteins.

At first glance, the overall spectral patterns of purified ribosomal proteins and WC-MS appeared considerably different, but there were peaks in common, most of which could be attributed to ribosomal proteins. Although the further identification of detected peaks is of interest, the spectral pattern in WC-MS was sufficient to use as a fingerprint for strain identification and classification.

### Clustering Analysis Using *Methylobacterium*-type Strains and Concordance between WC-MS &16S rRNA Sequence Similarity

**Figure 1:**
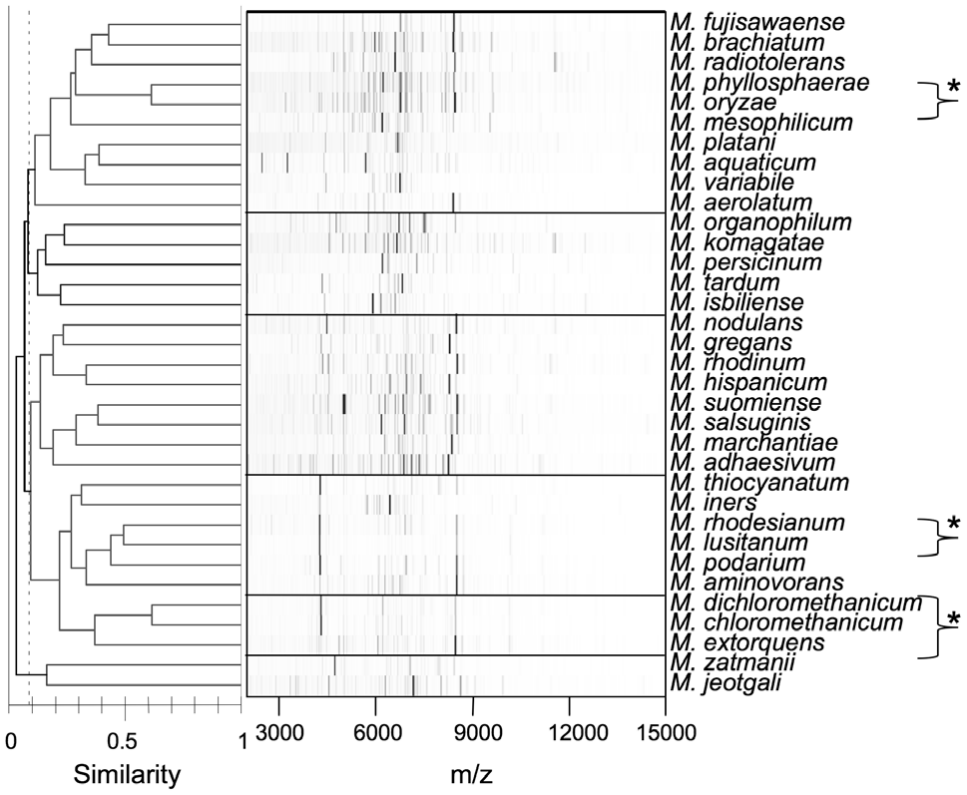
WC-MS profiles of the type strains of *Methylobacterium* species, and dendrogram calculated by our method. The spectra (m/z 2000–15,000) of relative intensity are shown as gel-like images using mMass 5.0 software [49]. Asterisks indicate the type strains showing similar spectra and are discussed in the text.

WC-MS data of the type strains of *Methylobacterium* cells grown on methanol were used to generate a distance matrix based on the Pearson correlation coefficient. Figure 1 shows WC-MS patterns of the type strains and calculated similarity shown as a dendrogram created by our Biotyper-independent method. Most of the type strains showed distinct spectral patterns, since they are genetically different. Some combinations (*M. oryzae* and *M. phyllosphaerae*, *M. extorquens* group and *M. rhodesianum* group, marked with asterisks) showed similar spectral patterns, which resulted in high spectral similarity values (approximately 0.5, as calculated by our method). This observation suggests that when one spectrum shows similarity of less than 0.5 to any of the type strains, it will be from a genetically novel species.

**Figure 2:**
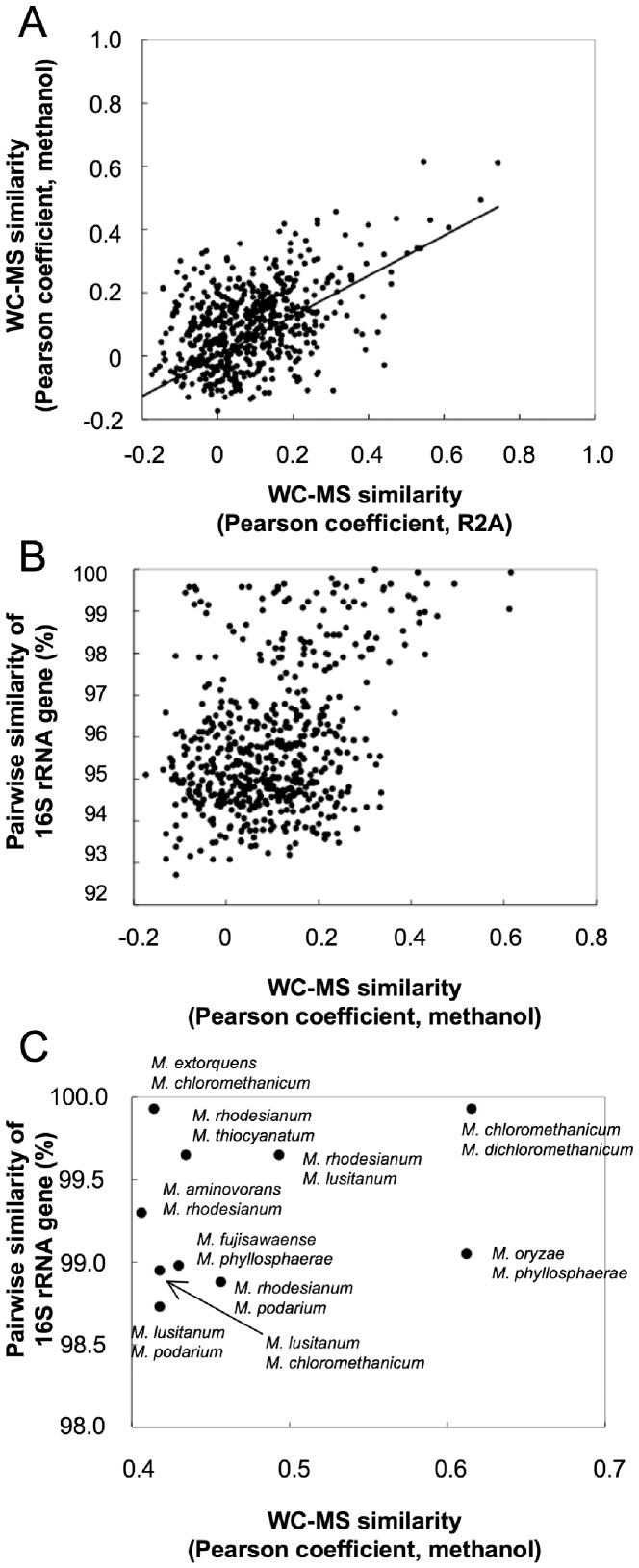
Correlation of the WC-MS data using different culture conditions and correlation of similarity between WC-MS data and 16S rRNA gene sequences. A. Mantel tests for similarities of WC-MS profiles of cells grown on R2A and methanol. B. Mantel tests for similarities of 16S rRNA gene and WC-MS on methanol. C. Enlarged image of B, showing combinations of the type strains sharing high 16S rRNA gene and WC-MS profile similarities.

Figure 2A shows a plot of similarity matrices based on WC-MS profiles of the type strains grown on R2A and methanol medium. The calculated correlation *p*-value was lower than 0.0001 in the Mantel test, suggesting that the matrices are significantly correlated and that the cultivation medium did not influence the resolution of species discrimination. Figure 2B shows a plot of similarity matrices based on pairwise similarity of the 16S rRNA gene and WC-MS using methanol-grown type strains. The Mantel test *p*-value was less than 0.0001, suggesting that the matrices are correlated. Plots above 97% [19] or 98.6% [20] indicated their high 16S rRNA gene similarity, even between type strains. Plots with high similarities in both the 16S rRNA gene and WC-MS were combinations of strains that differed in strain level (Fig. 2C). *M. dichloromethanicum* and *M. chloromethanicum* are synonyms of *M. extorquens*, and *M. lusitanum* is a synonym of *M. rhodesianum* [54]; they share high WC-MS similarities. *M. oryzae* and *M. phyllosphaerae* also showed high similarities, although their DNA-DNA relatedness is reported to be 8.06% [55]. Other plots included *M. thiocyanatum*, *M. aminovorans*, *M. fujisawaense*, *M. phyllosphaerae*, and *M. podarium*. They are close relatives in the phylogenetic tree based on the 16S rRNA gene [54], but they were shown to be different species by DNA-DNA relatedness analyses (less than 70%). One must be careful to discriminate the strains in this subgroup by WC-MS or their DNA-DNA relatedness may have to be re-examined.

Table S4 summarizes the calculated average molecular weight of ribosomal proteins in *Methylobacterium* species whose genome sequences are available. *M. extorquens* strains PA1 and AM1, *M*. *chloromethanicum* strain CM4, and *M. dichloromethanicum* strain DM4 share many identical ribosomal proteins, since they all belong to the *M. extorquens* group [54]. Among these, some proteins are also shared in *M. populi* and *M. radiotolerans*. *M. nodulans* and *Methylobacterium* sp. 4–46 also share some proteins of identical molecular weight, but they have no identical proteins with the *M. extorquens* group, *M. populi* (except for S20), and *M. radiotolerans*. Generally, the larger the ribosomal proteins are, the more differences their amino acid sequences contain, which results in less identical proteins with larger molecular weights. These observations suggested that there is no common ribosomal protein that should always be detected in all *Methylobacterium* species, i.e., there is no ribosomal biomarker that is specific to *Methylobacterium* species. This also supports the data showing that most of the type strains were highly discriminated in WC-MS analysis.

### Clustering Analysis of Isolates Using WC-MS

**Figure 3:**
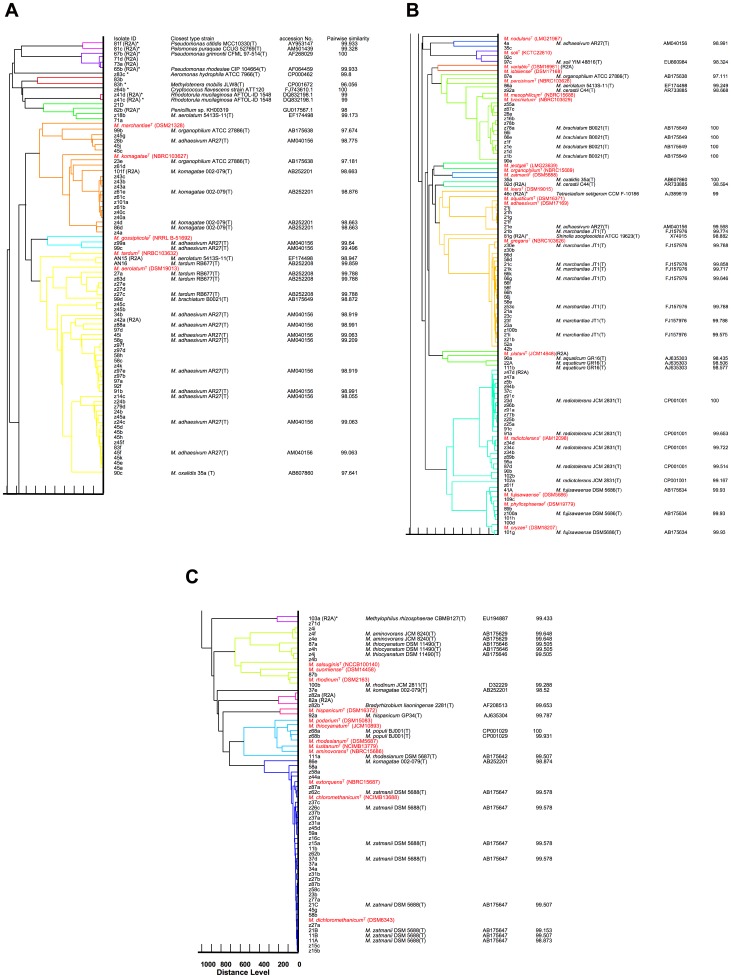
Biotyper-generated dendrogram based on WC-MS profile of the isolates. Non-*Methylobacterium* isolates are indicated with asterisks (*), which were revealed by 16S rRNA gene sequencing. The closest 16S rRNA gene relatives were determined by pairwise similarity analysis using the EZtaxon server [42]. Note that strains 23e and 35a were previously identified as novel species (*M. gnaphalii* 23e(T) and *M. oxalidis* 35a(T), respectively).

Clustering analysis based on WC-MS data of the isolates was carried out with MALDI Biotyper software (Bruker Daltonics). This software performs clustering analysis using a highly sophisticated pattern-matching algorithm. The result of the clustering analysis of 222 isolates (including those previously isolated, [56]) and all available type strains is shown in Fig. 3.

Most of the non-pigmented methylotrophs were clustered separately from *Methylobacterium* clusters. As discussed above, since the dendrogram does not necessarily reflect the evolutionary relationships, especially in the lower branch, some non-*Methylobacterium* species are also clustered in *Methylobacterium* clusters. In the dendrogram, *Methylobacterium-*type strains are well distinguished, except for the combination of *M. mesophilicum* and *M. brachiatum* (99.7% identity of the 16S rRNA gene), combinations of *M. fujisawaense*, *M. phyllosphaerae*, and *M. oryzae* (99.0–99.6%), and those of *M. podarium*, *M. thiocyanatum*, *M. rhodesianum*, *M. lusitanum*, and *M. aminovorans* (98.4–99.7%). They share a high percentage of 16S rRNA similarity, and their positions in the Biotyper-generated dendrogram were close. These results suggested either (i) that they should be considered the same species even though they were discriminated by previous studies or (ii) that while the core of their genomes, such as regions encoding ribosomal proteins are conserved, other accessory regions are not highly conserved.

*Methylobacterium* isolates from well-separated clusters were chosen for 16S rRNA gene analysis (ITS region was sequenced for fungi), and their identity is also shown in Fig. 3. Based on 16S rRNA gene homology searches, non-*Methylobacterium* methylotrophic isolates were related to *Pseudomonas otitidis, Pelomonas puraquae, Pseudomonas grimontii, Pseudomonas rhodesiae, Aeromonas hydrophila, Methylotenera mobilis, Cryotococcus flavescens, Rhodotorula mucilaginosa, Penicillium sp., Tetracladium setigerum, Shinella zoogloeoides, Methylophilus rhizosphaerae,* and *Bradyrhizobium liaoningense.* Most of them exhibited slow growth on methanol medium. Their methylotrophy needs to be studied in detail in the future, but this is not within the scope of the current study.

Isolates located near the type strains in the dendrogram exhibited high similarity of 16S rRNA gene to their corresponding type strains. This result suggested that WC-MS analysis could identify isolates without 16S rRNA gene sequencing. Isolates located in one of the biggest clusters of the *M. extorquens* group were identified as *M. zatmanii* by EZtaxon identification. *M. extorquens* and *M. zatmanii* share 99.6% 16S rRNA gene similarity. WC-MS analysis clearly distinguished these type strains, but identification based on pairwise similarity led to misidentification.

**Figure 4:**
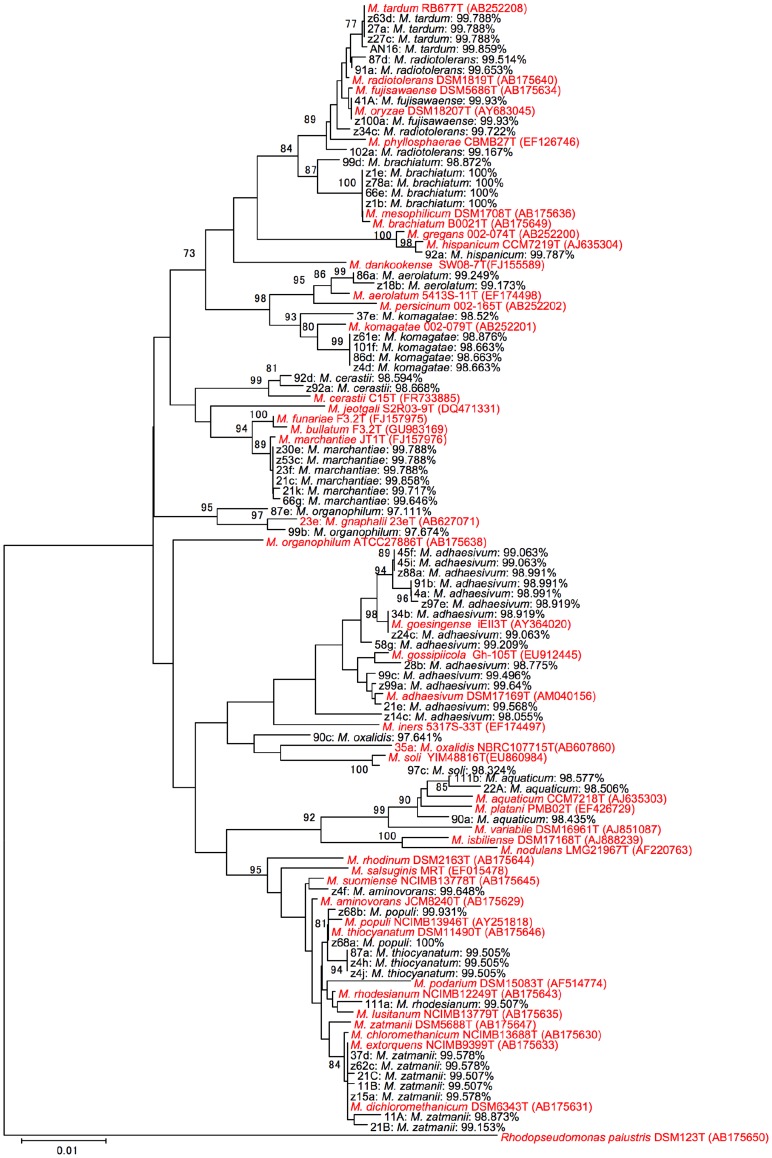
Neighbor-joining phylogenetic tree based on 16S rRNA gene sequences (ca. 1.44 kb) of the *Methylobacterium* isolates (black) and *Methylobacterium* type strains (red). Sequence alignment was carried out by SILVA Aligner [37] and analyzed with Kimura’s two-parameter algorithm (MEGA5) [38]. Tentative identifications by the EZtaxon site for the isolates are shown with pairwise similarity. Numbers at nodes are bootstrap percentages (based on 1000 resampled data sets); only values above 70% are shown. The sequence of *Rhodopseudomonas palustris* DSM123T (AB175650) was used as an outgroup. Note that strains 23e and 35a were previously identified as novel species (*M. gnaphalii* 23e(T) and *M. oxalidis* 35a(T), respectively). Bar: 0.01 substitutions per nucleotide position.

We generated a phylogenetic tree based on the sequences of the 16S rRNA genes of the *Methylobacterium* isolates (Fig. 4). Although we used a cultivation-dependent technique, isolates exhibited a wide range of phylogeny. Tentative identification by 16S rRNA gene sequencing was in good agreement with their positions to the closest type strains in the tree. However, isolates related to *M. radiotolerans* (e.g., z34c and 102a), *M. brachiatum* (99d), *M. komagatae* (37e), *M. organophilum* (87e, 23e, and 90c), *M. cerastii* (92d and z92a), *M. oxalidis* (90c), *M. adhaesivum* (45f, 45i, z88a, 91b, 4a, z97e, 34b, z24c, 58g, 28b, and z14c), *M. soli* (97c), and *M. aquaticum* (90a) showed relatively low identities of the 16S rRNA gene and a distant position to their closest type strains. Since their positions in the BioTyper tree were also distant from their corresponding type strains, they may represent new lineages of *Methylobacterium*. Thus, the selection of unique strains by WC-MS efficiently led to the finding of unique species. Although most of those listed above share high 16S rRNA gene similarity to their closest type strains (more than 97%), the clear difference in WC-MS profiles and distance in their positions in the phylogenetic tree strongly suggested their novelty. Indeed, strains 23e and 35a were genetically different from *M. organophilum* and *M. soli*, which exhibit the highest 16S rRNA gene similarity, respectively, and we previously proposed them as new species within the genus *Methylobacterium* (*M. gnaphali* [57] and *M. oxalidis* [44]), respectively.

### Chemotaxonomic Properties of Isolates

After the selection of unique strains in WC-MS and the sequencing of their 16S rRNA genes, we had a total of 190 strains of *Methylobacterium* species (plus 17 non-*Methylobacterium* isolates). Their phenotypic characteristics are summarized in Table S5. Overall, among the 190 *Methylobacterium* isolates, many strains were weakly positive in growth in the absence of an added nitrogen source, and 185 (methanol as a carbon source) and 93 (glucose as a carbon source) strains were positive in calcium phosphate solubilization. Siderophore production was positive in 35 strains. Average auxin production was 1.28 µg/ml, and average PQQ production was 4.6 µg/ml. The characteristics of the selected 80 strains for 16S rRNA sequencing were further examined. Most of the tested *Methylobacterium* isolates showed a negative or weak capability to utilize the tested sugars (with the exception of arabinose, in which 30 out of 66 isolates were positive). Urease was positive in 32 isolates. All isolates were negative in the DNase test and 11 strains were positive in the oxidase test. We did not find any correlation between 16S rRNA gene similarity and these phenotypic characteristics (data not shown).

Nitrogenase genes are encoded in the genomes of *M. nodulans and Methylobacterium* sp. 4–46, but not in those of other *Methylobacterium* species. Since plate tests are usually erratic, the presence of the *nifH* gene was confirmed by PCR [46]. We detected an amplified PCR product from *M. nodulans* as a positive control, but not from our isolates (data not shown), suggesting that no isolates possess nitrogenase genes. Their weak growth on nitrogen-free medium may be due to an air-borne compound containing nitrogen. Since it has been reported that the expression of glutamine synthetase in phyllospheric bacteria is prominent and no proteins for dinitrogen fixation ability were found in a metaproteomics study [58], the *Methylobacterium* species may be able to access nitrogen sources such as ammonia or amino acids and may not have to contain nitrogenase genes in the phyllosphere, except for nodulating *M. nodulans*. Calcium phosphate can be dissolved by gluconic acid, which is produced by PQQ-dependent glucose dehydrogenase [59]. The positive results with methanol as a carbon source may suggest another acidic substance, or acidic phosphatase activity, as reported earlier [60], although this was not further characterized in this study. A siderophore synthetase component (LucA/LucC family protein) is found in the genomes of *M. extorquens* strains AM1, PA1, DM4, and CM4, and *M. populi* but not in others, suggesting the dispersed distribution of the trait in the genus. Auxin is synthesized indigenously in plants, and also in plant-pathogenic, commensal, and symbiotic bacteria. The average value of auxin concentration in this study (1.28 µg/ml) was slightly lower than that previously reported for *M. extorquens* strain AM1 (6.1 µg/ml) [12]. Non-pigmented methylotrophic isolates, such as *Pseudomonas* isolates, had higher capability of synthesizing auxin (Fig. S2A). We are currently establishing a strategy to measure all phytohormones, including their derivatives synthesized by *Methylobacterium* species, using liquid chromatography-mass spectrometry. PQQ was recently reported to be an efficient plant growth promotion factor [61], and many gram-negative bacteria produce PQQ. In our survey, a variety of *Methylobacterium* species was found to produce PQQ, and some of them produced up to around 25 µg/ml PQQ (Fig. S2B). It is unknown whether PQQ is also one of the plant growth-promoting factors from the *Methylobacterium* species, but PQQ synthesis is likely essential for their colonization because of its necessity for methanol dehydrogenases [62]–[63]. We could not find any correlation between production levels of auxin and PQQ (data not shown), which suggested that these traits are not necessarily cooperating in the phyllospheric *Methylobacterium* species.

### Species Level Specificity between Plant and *Methylobacterium* Interaction

Based on 16S rRNA gene sequencing and the BioTyper-generated tree, the identity of the 191 *Methylobacterium* isolates and their isolation source is summarized in Fig. S3. The classification of bryophytes is based on Iwatsuki [64]. The angiosperm plants are classified based on Angiosperm Phylogeny Group III [65]. Isolates belonging to *M. exorquens, M. adhaesivum, M. marchantiae, M. komagatae, M. brachiatum,* and *M. radiotolerans* were found to be the most effective phyllospheric colonizers, as evidenced by the number of isolates and the diversity of isolation sources. Among them, most of the isolates that are phylogenetically close to *M. adhaesivum* are considered to be new lineages, and these isolates should be investigated for further characterization. Many strains that are close to the species have also been isolated from plants in a previous study [8]. Interestingly, 18 out of 57 isolates from bryophytes are considered to be of new lineages. Although many *Methylobacterium* species have been isolated from various plants, only some are from mosses (*M. marchantiae* [22], *M. funariae* [31], and *M. bullatum* [66], but the latter two share more than 99% 16S rRNA gene similarity with the former). Since bryophytes also emit methanol [56] and there are many bryophyte species that are adapted to extreme environments, they may be interesting isolation sources of novel bacteria. Indeed, a high diversity of *Methylobacterium* species observed by a culture-independent technique has been reported [23]. We could not observe any evolutionary relationship between the phylogenies of *Methylobacterium* species based on 16S rRNA gene and plant classification. This may be partly due to the limited number of isolates in this study. Alternatively, the evolution of *Methylobacterium* species may not have been dependent on that of plants.

In conclusion, we established a WC-MS-based technique for rapid classification of *Methylobacterium* species and demonstrated its effectiveness at distinguishing similar species, although there were some exceptions for genetically similar type strains. Since many of the detected peaks could be attributed to ribosomal proteins with some post-translational modifications, the spectral difference reflects the diversity and difference of the species, but not necessarily their evolutionary relationships. Sequencing-based classification is necessary to investigate evolutionary relationships, after the selection of unique strains in WC-MS analysis. The finding of novel species and identification of unknown isolates is quite easy with the WC-MS technique, since the analysis can be automated (several hundred samples a day) and there is no PCR step. This analysis would be the fastest among other PCR-based techniques for cultivated isolates. Based on the WC-MS-based dendrogram, we selected isolates and their phylogenetic attribution was clearly demonstrated, as evidenced by 16S rRNA gene sequencing. Some species were found to be effective and wide colonizers on plants. We found many strains of new lineages, especially from bryophytes. The evolutionary relationship between plants and *Methylobacterium* species could not be sufficiently clarified with our limited number of isolates. To address this, it is necessary to use the same plant species grown in different environments or to use different plant species in the same environments. Both of these parameters affect the *Methylobacterium* community composition [8], but it remains unknown what determines the species-level specificity of *Methylobacterium* residing in a specific plant. The cataloging of data for such interaction specificity will be necessary. We believe that this work with a wide variety of plant species can contribute to some extent. A number of possible novel species in the genus have been reported so far and await description. These studies also assist in the investigation of plant–microbe interaction specificity, especially for the *M. adhaesivum* group isolated in our study. Based on the results obtained in this study, a description for novel species ubiquitously colonizing on plants is necessary.

## Supporting Information

Figure S1 **WC-MS patterns of** ***M. extorquens*** **strain AM1 grown under different growth conditions for 2 weeks.** The informative range of spectra (m/z 3000–12,000) of relative intensity is shown as gel-like images using mMass 5.0 software [49]. Samples of 1- and 2-day cultures using methanol medium were not obtained due to poor growth.
(TIF)

Figure S2 **Production of auxin and PQQ by isolates.** The production levels of auxin (A) and PQQ (B) are shown with only 50 isolates of the highest production capability.
(TIF)

Figure S3 **Relationship between the identity of** ***Methylobacterium*** **isolates and their isolation sources.** The list of *Methylobacterium* is in the order of the phylogenetic tree constructed based on 16S rRNA gene sequences, as shown on the left. The tree was made in the same manner as that in Fig. 4. The number of isolates is shown in the table; parentheses indicate possible new lineages.
(TIF)

Table S1 **Plants used to isolate methylotrophs.**
(XLS)

Table S2 **m/z of detected peaks of the purified ribosome fraction and identification of proteins of** ***M. extorquens*** **strain AM1.**
(XLS)

Table S3 **m/z of detected peaks in WC-MS of strain AM1 and identification of proteins.**
(XLS)

Table S4 **Average molecular weight of ribosomal proteins in** ***Methylobacterium*** **species whose genome sequences are available.**
(XLS)

Table S5 **Characterization of isolates.**
(XLS)
