# Supplementary figures and images for: High-Throughput Identification and Screening of Novel Methylobacterium Species Using Whole-Cell MALDI-TOF/MS Analysis
Source: PLoS One. 2012 Jul 12;7(7):e40784. doi: 10.1371/journal.pone.0040784 (PMC3395638; doi:10.1371/journal.pone.0040784)

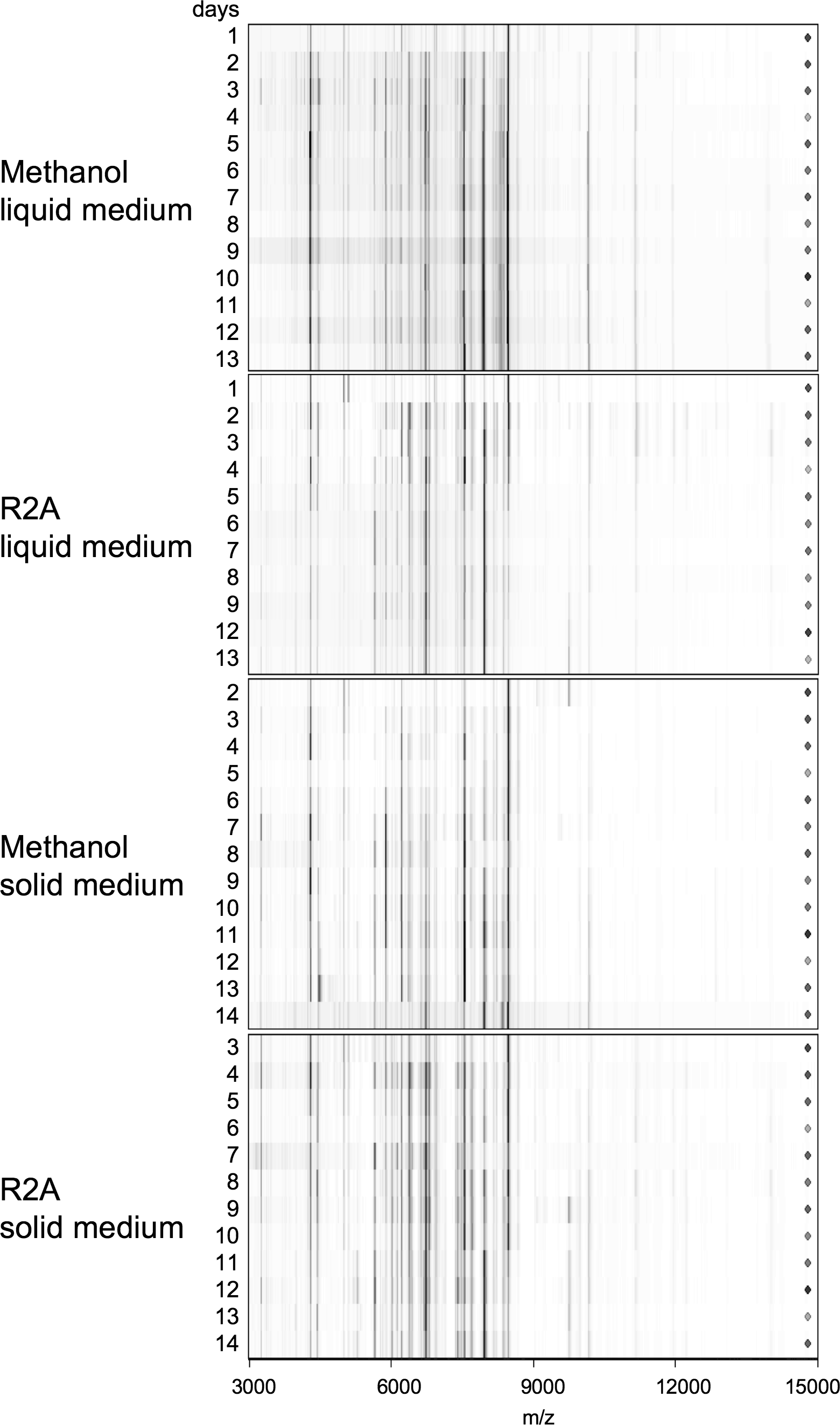

Supplement: Figure S1 — WC-MS patterns of M. extorquens strain AM1 grown under different growth conditions for 2 weeks. The informative range of spectra (m/z 3000–12,000) of relative intensity is shown as gel-like images using mMass 5.0 software [49]. Samples of 1- and 2-day cultures using methanol medium were not obtained due to poor growth. (TIF) [file pone.0040784.s001.tif]

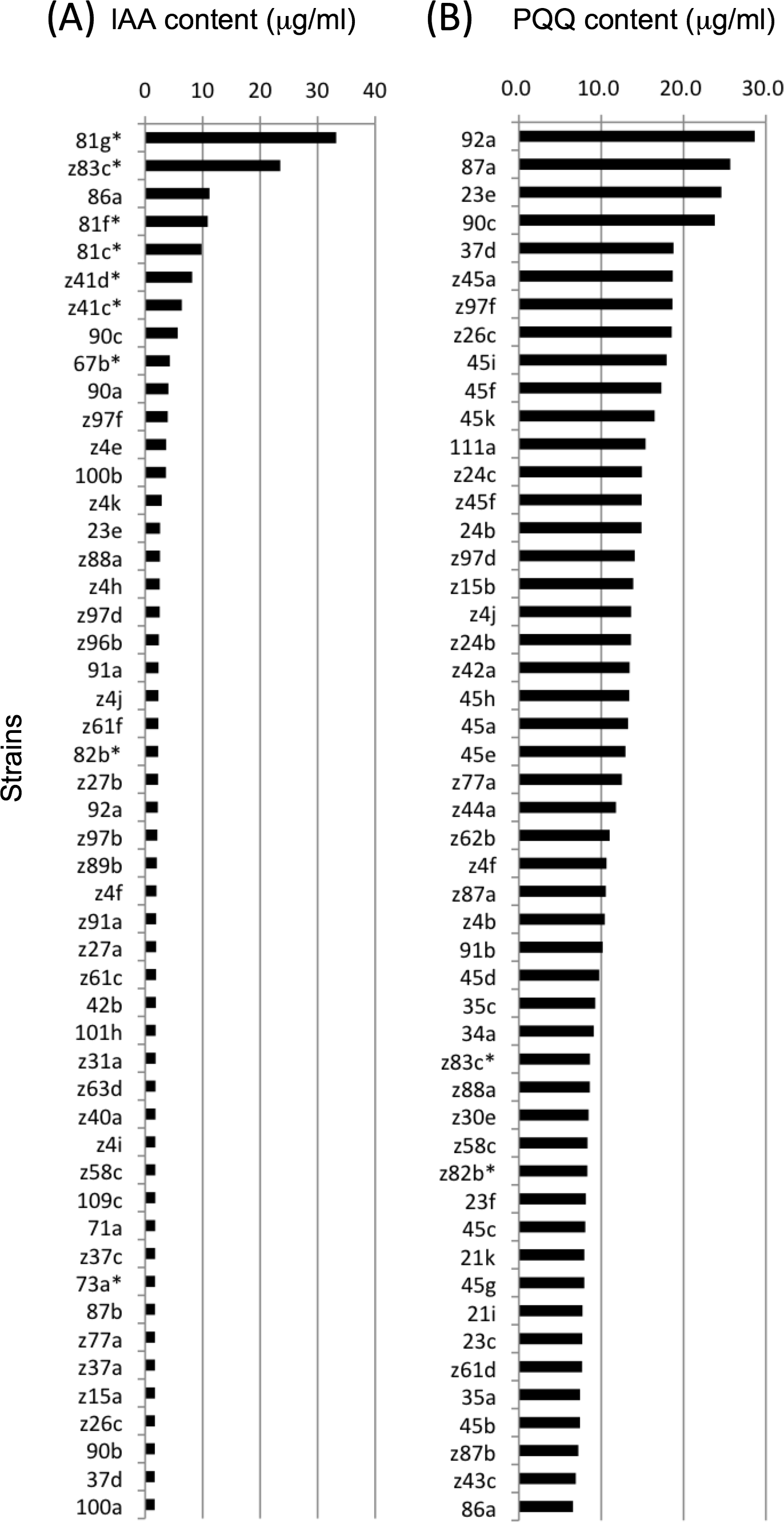

Supplement: Figure S2 — Production of auxin and PQQ by isolates. The production levels of auxin (A) and PQQ (B) are shown with only 50 isolates of the highest production capability. (TIF) [file pone.0040784.s002.tif]

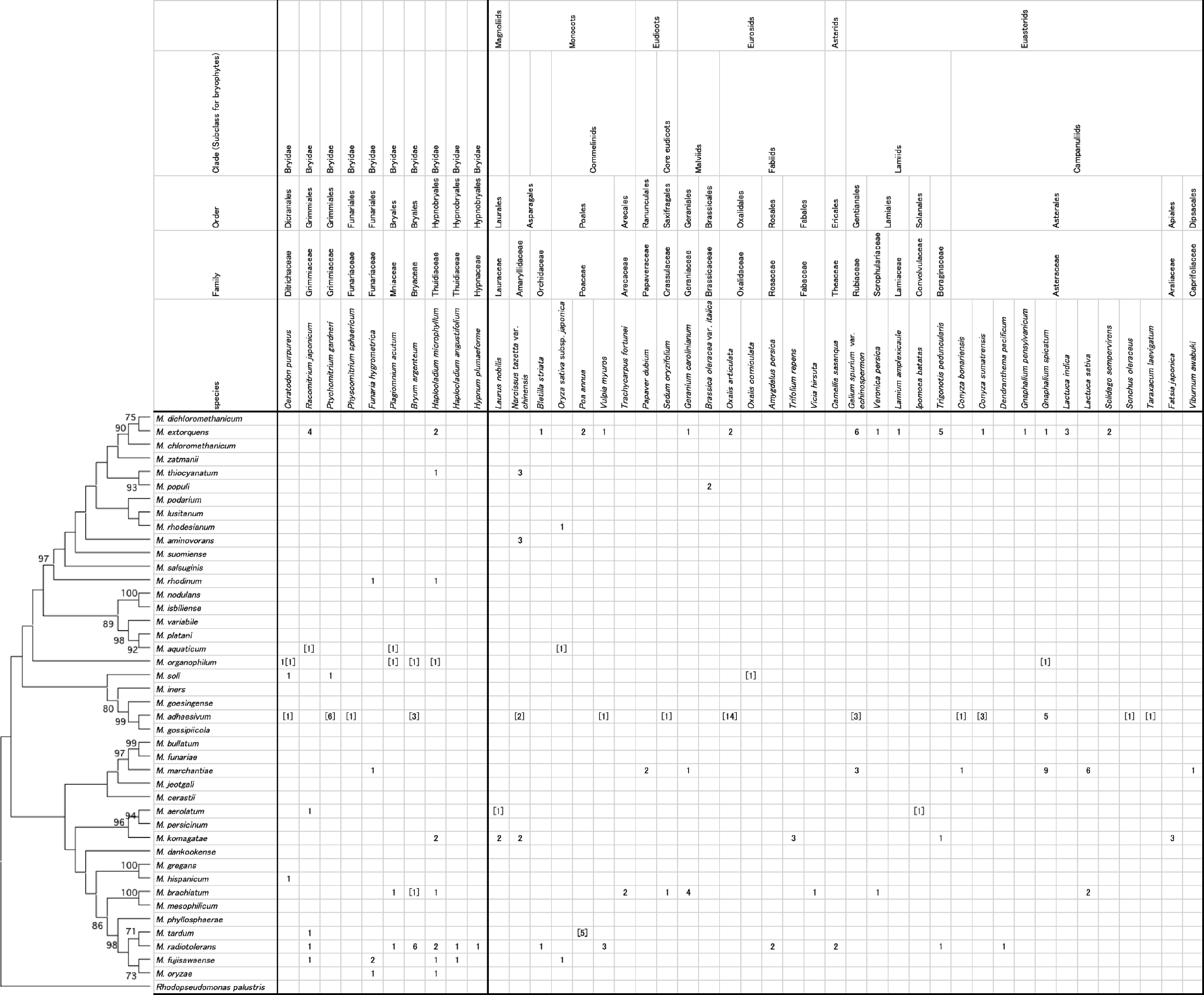

Supplement: Figure S3 — Relationship between the identity of Methylobacterium isolates and their isolation sources. The list of Methylobacterium is in the order of the phylogenetic tree constructed based on 16S rRNA gene sequences, as shown on the left. The tree was made in the same manner as that in Fig. 4. The number of isolates is shown in the table; parentheses indicate possible new lineages. (TIF) [file pone.0040784.s003.tif]
